# Genetically encoded sensors enable micro- and nano-scopic decoding of transmission in healthy and diseased brains

**DOI:** 10.1038/s41380-020-00960-8

**Published:** 2020-12-04

**Authors:** Li Lin, Smriti Gupta, W. Sharon Zheng, Ke Si, J. Julius Zhu

**Affiliations:** 1 Department of Neurosurgery, the First Affiliated Hospital of Wenzhou Medical University, Wenzhou, 325035 China; 2 School of Pharmaceutical Sciences, Wenzhou Medical University, Wenzhou, 325035 China; 3 Department of Pharmacology, University of Virginia School of Medicine, Charlottesville, VA 22908 USA; 4 Biomedical Engineering Class of 2021, University of Virginia School of Medicine, Charlottesville, VA USA; 5 College of Optical Science and Engineering, Zhejiang University, Hangzhou, 310027 China; 6 School of Brain Science and Brain Medicine, Zhejiang University, Hangzhou, 310027 China

**Keywords:** Biotechnology, Diseases

## Abstract

Neural communication orchestrates a variety of behaviors, yet despite impressive effort, delineating transmission properties of neuromodulatory communication remains a daunting task due to limitations of available monitoring tools. Recently developed genetically encoded neurotransmitter sensors, when combined with superresolution and deconvolution microscopic techniques, enable the first micro- and nano-scopic visualization of neuromodulatory transmission. Here we introduce this image analysis method by presenting its biophysical foundation, practical solutions, biological validation, and broad applicability. The presentation illustrates how the method resolves fundamental synaptic properties of neuromodulatory transmission, and the new data unveil unexpected fine control and precision of rodent and human neuromodulation. The findings raise the prospect of rapid advances in the understanding of neuromodulatory transmission essential for resolving the physiology or pathogenesis of various behaviors and diseases.

## Introduction

Dysfunction in neural communication is the primary cause of a plethora of psychiatric and neurological disorders. Large genetic association studies implicate that structural and functional alterations in fast transmission mediated by fast-acting transmitters, i.e., glutamate and gamma-aminobutyric acid (GABA), may be responsible for a common underlying pathology in many symptomatically distinct cognitive disorders [1, 2]. Corroborating this view, electrophysiological and other functional studies have identified various aberrant synaptic mechanisms underlying the pathogenesis of diseases [3, 4]. Neuromodulation research has shown that slow transmission mediated by slow-acting transmitters, such as acetylcholine (ACh), monoamines, and neuropeptides, participates in myriad physiological and pathological processes. Likewise, dysregulation of slow neuromodulatory transmissions is associated with major brain disorders, including addiction, Alzheimer’s disease, autism, cardiovascular diseases, depressive disorders and schizophrenia, eating disorders, epilepsy and sleep disorders [5–10]. However, in contrast to research on glutamatergic and GABAergic neurotransmission, delineating synaptic regulation and dysregulation of neuromodulatory transmissions remains a daunting task, which has hampered efforts in mechanistically understanding the neuromodulation-related behaviors and diseases.

The slow advance in defining transmission properties of neuromodulatory communication is due primarily to limitations of currently available tools for monitoring neuromodulatory transmissions. Patch-clamp recordings, which can make reliable repetitive measurements of prominent current responses of fast transmission, serve as the prime method to interrogate fundamental synaptic properties of glutamatergic and GABAergic transmission [11, 12]. However, small and rapidly desensitizing neuromodulatory responses make patch-clamp recordings or electrophysiology ineffective in demarcating synaptic parameters of neuromodulatory transmission [13, 14]. The other currently available neuromodulatory transmission monitoring tools do not work well for this problem, either. For instance, although the detection sensitivity of microdialysis, a frequently employed method, has been improved in recent years, the poor spatial and temporal resolution still limits its ability to assess dynamics of cholinergic and monoaminergic signals [15, 16]. Fast scan cyclic voltammetry provides excellent nanomolar sensitivity and millisecond temporal resolution, but this detection approach is set back by its poor spatial resolution and inability to distinguish norepinephrine (NE) and dopamine (DA) [17]. Recent effort has led to the development of Förster Resonance Energy Transfer- and cell-based fluorescent ACh and monoamine sensors [18, 19]. However, the low-sensitivity and/or low-resolution of these sensors permits detection of only volume-size transmission, precluding their application in resolving synaptic properties of neuromodulatory transmission. The limitations, nevertheless, inspire a greater desire to engineer user-friendly and broadly applicable genetically encoded neurotransmitter sensors that permit tissue-specific high-resolution measurements of neuromodulatory transmission [20, 21].

### Genetically encoded neuromodulatory transmitter sensors

**Fig. 1:**
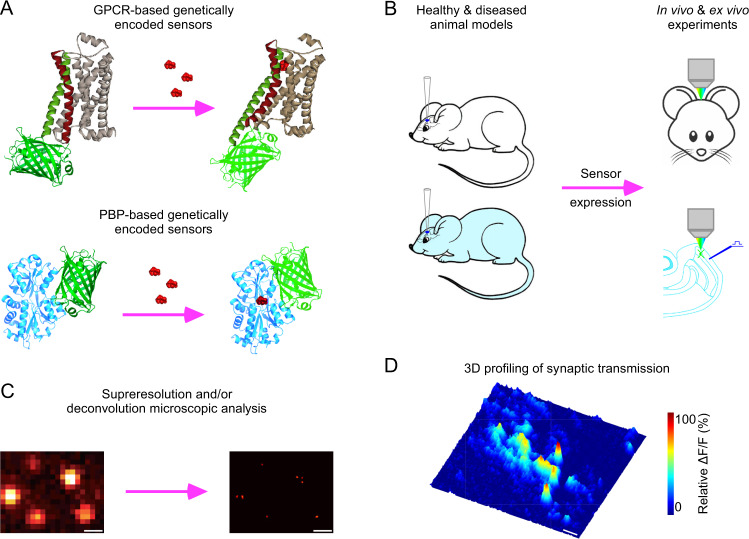
High-resolution analysis of genetically encoded sensor-illustrated transmission. **A** Schematic of G protein-coupled receptor (GPCR)- and bacterial periplasmic binding protein (PBP)-based genetically encoded sensors for neuromodulatory transmitters. **B** Schematic of viral expression, in vivo and ex vivo applications of genetically encoded sensors. **C** Schematic of superresolution and/or deconvolution microscopic analysis of image data obtained with genetically encoded sensors. **D** Three-dimensional spatiotemporal profiling of synaptic transmission. Note the collection of exemplary animal experimental data made with the recently published project [32].

Recently, colossal collective efforts of tool engineers and biologists yielded several intensity-based genetically encoded sensors for ACh and monoamines (Fig. 1). These sensors consist of a conformationally sensitive circularly permutated GFP (cpGFP) and a ligand-binding protein that alters cpGFP fluorescence by inducing conformational changes upon transmitter binding (Fig. 1A). Two major groups of genetically encoded transmitter sensors were created: G protein-coupled receptor (GPCR)- and bacterial periplasmic binding protein (PBP)-based sensors. GPCR-based sensors, which have the third intracellular loop of primogenitor GPCRs replaced with a cpGFP, frequently inherit the excellent membrane surface trafficking, ligand-binding affinity and pharmacological properties from their primogenitor GPCRs, and are often ready to detect endogenously released transmitters [22, 23]. However, these sensors may have limited dynamics and/or slow kinetics due to the primogenitors’ slow kinetics and limited conformational changes associated with ligand binding [24–26]. Because PBPs have lower affinities with their ligands, creating the PBP-based sensors with sensitivity matching the endogenous transmitter levels may require painstaking evolution, yet the effort can lead to high-performance sensors with fast kinetics and large dynamics [27–30]. Membrane surface trafficking of the PBP-based sensors can be less than optimal, but these sensors are usually more amenable for targeted expression in other subcellular compartments. Table 1 summarizes the properties of recently developed genetically encoded sensors for ACh and monoamines.

**Table 1:** Key properties of genetically encoded sensors for acetylcholine and monoamines.

Sensors (ref)	Sensitivity (µM)	*Δ*F/F (%)	SNR (puff)	Kinetics (ms)	Selectivity
ACh sensors					
GRAB_ACh2.0_ [24]	EC_50_ = ~1–2 µM	~90%	~14	*τ*_*on*_ = ~250 ms; *τ*_*off*_ = ~700 ms	High
GRAB_ACh3.0_ [31]	EC_50_ = ~2 µM	~280%	–	*τ*_*on*_ = ~100 ms; *τ*_*off*_ = ~900 ms	High
iAChSnFR [29]	*K*_d_ = ~1 µM	~1200%	~50	*k*_on_ = 0.62 μM^−1^s^−1^; *k*_off_ = 0.73 s^-1^	*K*_d_ = ~45 µM (choline)
NE sensors					
GRAB_NE1m_ [33]	EC_50_ = ~2 µM	~230%	~75	*τ*_*on*_ = ~70 ms; *τ*_*off*_ = ~700 ms	EC_50_ = ~1400 µM (DA)
GRAB_NE1h_ [33]	EC_50_ = ~0.1 µM	~130%	~10	*τ*_*on*_ = ~35 ms; *τ*_*off*_ = ~2000 ms	EC_50_ = ~0.6 µM (DA)
5HT sensors					
GRAB_5HT0.5_ [34]	EC_50_ = ~0.25 µM	~280%	~50	*τ*_*on*_ = ~60 ms; *τ*_*off*_ = ~3300 ms	High
GRAB_5HT1.0_ [34]	EC_50_ = ~0.02 µM	~250%	–	*τ*_*on*_ = ~200 ms; *τ*_*off*_ = ~3000 ms	High
iSeroSnFR [35]	EC_50_ = ~1.5 µM *K*_d_ = ~350 µM	~1000%	–	*τ*_*fast*_ = ~4 ms; *τ*_*slow*_ = ~100 ms	High
DA sensors					
GRAB_DA1m_ [25]	EC_50_ = ~0.13 µM	~90%	–	*τ*_*on*_ = ~60 ms; *τ*_*off*_ = ~700 ms	EC_50_ = ~1.5 µM (NE)
GRAB_DA1h_ [25]	EC_50_ = ~0.01 µM	~90%	–	*τ*_*on*_ = ~140 ms; *τ*_*off*_ = ~2500 ms	EC_50_ = ~0.1 µM (NE)
GRAB_DA2m_ [36]	EC_50_ = ~0.1 µM	~340%	–	*τ*_*on*_ = ~40 ms; *τ*_*off*_ = ~1300 ms	EC_50_ = ~1.2 µM (NE)
GRAB_DA2h_ [36]	EC_50_ = ~0.03 µM	~280%	–	*τ*_*on*_ = ~50 ms; *τ*_*off*_ = ~7300 ms	EC_50_ = ~0.07 µM (NE)
rGRAB_DA1m_ [36]	EC_50_ = ~0.1 µM	~150%	–	*τ*_*on*_ = ~80 ms; *τ*_*off*_ = ~770 ms	EC_50_ = ~2.2 µM (NE)
rGRAB_DA1h_ [36]	EC_50_ = ~0.02 µM	~100%	–	*τ*_*on*_ = ~60 ms; *τ*_*off*_ = ~2150 ms	EC_50_ = ~0.06 µM (NE)
dLight_1.1/1.2_ [26, 37]	*K*_d_ = ~0.3–0.7 µM	~300%	–	*τ*_*on*_ = ~10 ms; *τ*_*off*_ = ~100 ms	*K*_d_ = ~20 µM (NE)
dLight_1.3_ [26, 37]	*K*_d_ = ~2.0 µM	~900%	–	–	–
RdLight_1_ [41]	*K*_d_ = ~2.0 µM	~250%	–	*τ*_*on*_ = ~15 ms; *τ*_*off*_ = ~400 ms	*K*_d_ = ~20–100 µM (NE)

Genetically encoded ACh sensors include GPCR-based GRAB_ACh2.0_ and GRAB_ACh3.0_ [24, 31], and PBP-based iAChSnFRs with both green and yellow fluorescent versions [29] (Table 1). Directly comparing the perforamance of GRAB_ACh_ and iAChSnFR sensors in the same medial entorhinal cortical preparation revealed a few key differences between these two families of sensors [29, 32]. Although GRAB_ACh_ sensors produced robust fluorescence responses, their inherited slow kinetics significantly attenuated and delayed high-frequency cholinergic signals; they were excellent binary cholinergic signal detectors [24, 31]. iAChSnFRs performed much better in faithfully following cholinergic signals in the entire frequency range [29, 32]. Although the fluoresence response ΔF/F of iAChSnFRs is less than optimal, the sensors do have a large dynamic response range for improvement in fluorescence responses [29].

Two GPCR-based norepinephrine (NE) sensors, GRAB_NE1m_ and GRAB_NE1h_, are currently available (Table 1), with GRAB_NE1m_ performing better in detecting endogenous adrenergic signals [33]. We expressed iAChSnFR and GRAB_NE1m_ in the same amygdalar preparation to compare their ability in measuring endogenous signals and the results indicated that GRAB_NE1m_ did not rival iAChSnFR in speed and fluorescence response size [32]. Nevertheless, GRAB_NE1m_ could still follow the relatively slower adrenergic transmission ([32]; our unpublished data). These results raise the hope that GRAB_NE1m_ may resolve basic transmission properties of adrenergic modulation, even though the experiments could be technically challenging due to the small adrenergic fluorescence responses [32].

There are both GPCR and PBP-based genetically encoded sensors for serotonin (5-hydroxytryptamine or 5HT) (Table 1) [34, 35]. GPCR-based GRAB_5HT0.5_ and GRAB_5HT1.0_ effectively detected serotonergic signals in populations of cells [34]. GRAB_5HT0.5_ performed better in assessing endogenous single-cell serotonergic signals and could serve as a viable tool for dissecting transmission properties of serotonergic modulation, due presumably to its kinetics and affinity matching more closely to the dynamics and concentration of endogenously released 5HT [32, 34]. The PBP-based iSeroSnFR reported prominent endogenous serotonergic signals in populations of cells [35]. However, given its relatively lower sensitivity (compared to GRAB_5HT_) [34, 35], it waits to see whether the sensor can detect single-cell and subcellular serotonergic signals.

Two families of GPCR-based dopamine (3,4-dihydroxyphenethylamine or DA) sensors, human D_2_ dopamine receptor-based GRAB_DA_ [25, 36] and human D_1_ dopamine receptor-based dLight [26, 37] sensors, are engineered (Table 1). GRAB_DA1m_, GRAB_DA1h_, GRAB_DA2m_, and GRAB_DA2h_ are the green versions of genetically encoded DA sensors, while rGRAB_DA1m_ and rGRAB_DA1h_ belong to the first red versions of genetically encoded neuromodulatory transmitter sensors [25, 36]. dLight sensors also have multiple versions with somewhat different properties [26, 37]. The top-performing GRAB_DA2m_ and dLight_1_ registered robust dopaminergic signals from populations of cells in the striatum [32, 36] and basal amygdala [38], which receive the heaviest dopaminergic innervations with probably largest dopaminergic signals [38–40]. A recent addition is a D_2_ dopamine receptor-based red version of genetically encoded DA sensor, RdLight_1_ [41], further expanding the toolbox for DA sensors. Direct experimental comparison showed that GRAB_DA2m_ produced slightly larger single-cell dopaminergic responses with a better signal-to-noise ratio compared to dLight_1_ in the same striatal preparation [32, 36]. While the extremely low baseline dLight_1_ fluorescence often precludes identification of individual expressing cells, dLight_1_ appears to have slightly higher selectivity over NE than GRAB_DA2m_ [26, 36]. It remains unclear whether GRAB_DA2m_ and dLight_1_ are capable of detecting modest single-cell dopaminergic signals at other brain areas, such as the prefrontal cortex and hippocampus.

### A new sensor-based analysis method

The development of genetically encoded transmitter sensors offers opportunities to make groundbreaking advances in neuromodulation research. While many researchers use the new sensors merely as sensitive detectors to monitor dynamic neuromodulatory signals, as advocated by recent reviews [22, 23, 42, 43], these sensors could actually do more than that. The new genetically encoded neuromodulatory sensors, fortuitously, emit a large amount of photons with their fluorescence responses being largely independent of the expression levels [29, 32–34]. The welcoming properties, when combined with the recent advances of superresolution and/or deconvolution microscopy [44, 45], make it possible to quantify transmission properties of neuromodulation [11], which is essential for demarcating the physiology of behaviors and the pathogenesis of diseases [1–4].

Initial experiments demonstrated that top-performing sensors could resolve synaptic properties of neuromodulatory transmission [29, 32–34]. There are, unfortunately, a significant number of recently published “proof-of-principle” tools or methods that turned out to be biophysically uninterpretable, methodologically unreproducible, and/or biologically inapplicable [46, 47]. To rigorously introduce this genetically encoded neurotransmitter sensor-based superresolution and deconvolution microscopic analysis method, we here present its biophysical foundation, practical solutions, biological validation, and broad applicability.

### Biophysical foundation

From the 16th to 19th centuries, the trial-and-fail craftsmanship is the primary means to improve microscopic images. In the late 19th century, the Ernst Abbe’s arduous computational work, corroborated by experiments carried out in the Carl Zeiss workshop, demonstrated that only knowing the exact light behavior can one gain insight into and control over all decisive factors influencing the microscopic performance [48]. This work led Abbe to conclude that wave optics and diffraction posed fundamental limits on the ability to image, or the Abbe limit of resolution [49]. It gives the minimum distance ∆*x* between two distinguishable objects as

1 $$\Delta X_{{\mathrm{Abbe}}} = {\uplambda}/\left( {2n \bullet {\mathrm{Sin}}\alpha } \right) \equiv {\uplambda}/2{\mathrm{NA}},$$

where *n* gives the index of refraction of the medium in which the image is taken, α is the maximum angle between the optical axis and all rays captured by the microscope objective, and NA stands for numerical aperture used to describe the resolution of microscope.

Later, many researchers revisited the concept of resolution limit, and proposed that in principle, cooperative captivation of many photons could increase resolution beyond the Abbe diffraction limit [50, 51]. In practice, only in the last ~20 years, the rapid instrumental and biological advances result in numerous feasible applications, particularly in biology, that routinely surpass of the diffraction limit, a phenomenon termed “superresolution.” A number of high-resolution imaging tools including superresolution microscopy, light-sheet microscopy, structured illumination microscopy, confocal microscopy, multiphoton microscopy, deconvolution microscopy, and others, have emerged. Many of them may be classified into two major groups: superresolution microscopy and deconvolution microscopy, where the others are hard to classify into either group because they contain properties of both [52, 53].

Through the hardware optimization, superresolution microscopy (e.g., photo-activated localization microscopy, stochastic optical reconstruction microscopy, and stimulated emission depletion microscopy) can achieve excellent resolution improvement with increasing photon counting, following the equation as

2 $$\Delta X_{{\mathrm{superresolution}}\,{\mathrm{microscopy}}}\sim \Delta X_{{\mathrm{Abbe}}} \bullet N^{ - 1/2},$$

where *N* represents the number of photons detected. This favorable resolution scaling allows superresolution microscopy to fundamentally surpass the classical diffraction limit and reach the sub-100-nm resolution range in most applications, with the possibility of achieving ~10-nm resolution, or down to the single-molecule resolution [45, 52, 54]. The tradeoff of superresolution microscopy is imaging speed, particularly in applications of wide-field living imaging that desires high temporal resolution. In addition, superresolution microscopic procedures often discard data from crowded molecules with overlapping images, wasting valuable image information partly degraded by overlapping.

Deconvolution microscopy, which uses computational procedures to improve the quality of microscopic images, can be an excellent alternative for high-resolution living-cell imaging, despite its less favorable resolution scaling as

3 $$\Delta X_{{\mathrm{deconvolution}}\,{\mathrm{microscopy}}}\sim \Delta X_{{\mathrm{Abbe}}} \bullet N^{ - 1/4},$$

The rapidly increased applications of deconvolution microscopy attribute mainly to new genetic tools that yield more collectable photons and expeditiously improved computational algorithms [53, 55, 56]. Given that high-performance genetically encoded neurotransmitter sensors can achieve dense expression of fluorophores and emit large amounts of photons upon transmitter binding, it is possible to achieve wide-field living imaging of transmitter release at the ~100−200-nm resolution and resolve many fundamental synaptic properties of neurotransmission [32]. Of course, the unfavorable *N*^−1/4^ resolution scaling for deconvolution microscopy means that a modest increase in resolution requires a large increase in photon numbers, making the resolution improvement less economically favorable. The combined deconvolution-superresolution microscopic techniques, which may achieve both high-resolution and high-imaging speed, are excellent alternative approaches [57, 58].

The above-mentioned optical properties predict that genetically encoded neurotransmitter sensors can make reliable measurements of neuromodulatory transmitter releases with nano- and micro-scale spatiotemporal resolution. The measurements may offer reasonably accurate estimations of fundamental synaptic properties of neuromodulatory transmission, such as the transmitter diffusion extent, number of release sites, release pool size, release probability, quantal size, and refilling rate [11, 59]. Defining the synaptic parameters builds the scientific foundation for interrogation of the regulation, function, and short-term and long-term plasticity of neuromodulatory transmission, and sets the neuromodulation baseline for medication testing and development.

### Practical solutions

Following good practical solutions is central to accurate measurements of synaptic properties of neuromodulatory transmission, given particularly that many genetically encoded sensors remain to be optimized to suit them to this task. Selecting the top-performing sensors is the first key step. iAChSnFR, GRAB_NE1m_, and GRAB_5HT0.5_ detected robust but variable single-cell responses in every ex vivo and in vivo neuronal and non-neuronal tissues examined [29, 32–34], supporting their applicability in resolving transmission properties in general tissue preparations ([32]; our unpublished data). GRAB_DA2m_ and dLight_1_ could achieve the same task in the striatum [25, 26, 32, 36], but might not in the other brain areas ([32]; our unpublished data). The other sensors require further evaluation. Because the existing genetically encoded neuromodulatory transmitter sensors are developed by a very few rigorous research groups, one may deduce the relative performance of sensors generated from the same groups based on the reported sensor specificities (e.g., sensitivity, ΔF/F, SNR, and kinetics) (Table 1).

Expression of genetically encoded transmitter sensors is another key step, which can be achieved with various gene expression approaches, including the frequently employed viral expression systems (e.g., adeno-associated virus (AAV), lenti and Sindbis viruses) (Fig. 1B). Sindbis virus has the most efficient production time of ~1.5 days, shortest expression time of ~8–24 h, largest payload of up to 15 kb (ready for co-expression multiple transgenes) and highest expression levels (favored for high spatiotemporal image resolutions), albeit the shortest viable expression time of ~3–5 days [60–62]. In contrast, several serotypes of AAV viruses have been extensively used due to its minimal toxicity, persistent expression time of ≥6 months (preferred for long-term physiological and behavioral tests), and noninvasive delivery possibility [63, 64]. However, the small payload for transgene up to ~4.5–5 kb, low viral production efficiency and slow expression time of ≥3 weeks are the major limitations of AAV viruses. Lentivirus, which has relatively faster production, larger payload of up to ~8–10 kb, shorter-expression time of ~1–2 weeks, and long-lived expression of multiple months [65–67], offers a solution in compromise.

Image acquisition and analysis are the last two key steps. Given the advantages of low instrumental requirement and wide-field living imaging applicability, deconvolution microscopy is practically favored in many biological applications. Since the goal of deconvolution microscopic analysis is to “reassign” the light to its original place, the practical solutions shall focus on correction for image drift and fluctuations, minimization of photobleaching, auto fluorescence, and noise, and optimization of light diffraction correction, many of which are applicable to superresolution microscopy and combined deconvolution-superresolution microscopy.

Image drifts and fluctuations can lead to the significant artifactual reassignment of light. Thus, optimizing the imaging system to minimize the drift and fluctuations is worth the effort. The electrophysiological setups designed for stable multiple patch-clamp recordings are usually optimized to mitigate the experimental drift and fluctuations [68, 69], and they can easily be adapted to acquire stable living-cell images with the minimal drift and fluctuations readily correctable with an intensity-based registration function [32]. Photobleaching and auto fluorescence may result in incorrect estimation of real light intensity distribution and lead to errors in photon reassignment. Running correction algorithms can minimize photobleaching and auto fluorescence [32, 70]. Noise, a random process responsible for image degradation, comes from two main sources, including photon noise following Poisson distribution and electronic noise in Gaussian nature that is often negligible in modern detectors. Because noise noticeably limits the efficiency of deconvolution algorithms, including denoising procedures at various stages of deconvolution algorithms is highly desired.

Light diffraction through an optical system, which follows the description of its point spread function (PSF), induces optical blur that limits optical resolution. Deblurring is a key process of deconvolution microscopy that uses PSF to reverse optical distortion to “reassign” the light to its original place [71]. Therefore, obtaining the correct PSF, via either theoretical calculation or empirical measurement, is essential for the quality of restored images. Because theoretical PSF models apply only to perfect lenses and well-defined optical paths, and some spherical aberrations are difficult to predict theoretically, empirical measurements are preferred in practice. For accurate determination of an optical system’s PSF, it is best to measure PSF at the same system under the same conditions used for the image acquisition. One simple way to obtain PSF is to acquire images of commercially available fluorescent microspheres. In theory, the smallest beads work best. However, small beads are weaker in intensity and bleach more rapidly, and thus, in practice larger beads with a diameter smaller than half the resolution work just fine. To reduce the noise from the empirically measured PSFs, one may average measurements from multiple beads, multiple trials of single beads, and/or multiple circular axes of single beads [32]. It is important to note that deconvolving large datasets of imaging episodes of neuromodulatory transmissions is computationally intensive tasks, taking hours, if not days [32]. Hence, it is wise to employ the optimized and deep learning analysis algorithms to carry out these tasks [44].

### Biological validations

The sensor-based image analysis method has been used to resolve fundamental biological questions, which provides a rigorous validation of its applicability. For example, genetically encoded neurotransmitter sensors, which enable the first high-resolution visualization of spatial diffusion of endogenously released neuromodulatory transmitters [32] (Fig. 1C, D), was employed to resolve a long-standing biological question regarding the primary neuromodulatory transmission mode [72, 73]. The widely accepted theory of neuromodulatory transmission, proposed three decades ago, postulates that the primary mode of intercellular neuromodulatory communication is volume transmission that takes place among cells in general regions, rather than between specific cells that form direct circuits or contacts [74, 75]. This model specifically purports that ACh and monoamines diffuse into local areas, affecting many different types of nearby cells, and neuropeptides travel even farther, influencing both local cells and distant cells millimeters away [74, 76]. The foundation of the theory is based primarily on the assumption that endogenously released neuromodulatory transmitters behave similarly as exogenously applied ones (that diffuse more freely in the extrasynaptic space), which has not yet been corroborated by any direct experimental evidence [72, 73]. Genetically encoded neuromodulatory transmitter sensors, in combination with deconvolution microscopic analysis, permitted the first direct visualization of spatial diffusion of neuromodulatory transmitters at individual release sites, yielding spatial diffusion spread length constants of ~0.75 µm for both ACh and monoamines ([32]; cf. [77–79]) (Fig. 1C, D). High-speed two-photon imaging technique later verified the value for endogenously released ACh in the intact brain [29]. These results indicate that highly restricted, non-volume neuromodulatory transmission is a key mode for intercellular communication.

**Fig. 2:**
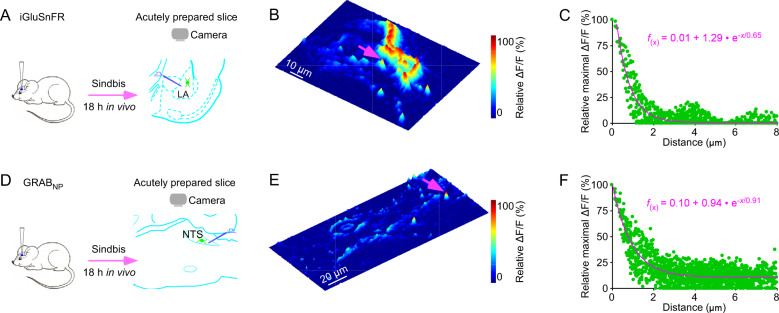
Visualizing spatial transmitter diffusion with genetically encoded sensors. **A** Schematic of stimulation-imaging experiments in acute mouse amygdalar slices. LA Lateral amygdala. **B** Three-dimensional spatiotemporal ΔF/F profiling of an iGluSnFR expressing amygdalar neuron to local electrical stimuli. Note one isolated release site indicated by pink arrow in **B**. **C** Pixel-wise maximal ΔF/F plot at the isolated release site indicated by the pink arrow in **B**. Fitting the data points in this plot with a single exponential decay function (pink line) yields an estimated glutamate spread length constant of 0.65 µm. Note the collection of exemplary animal experimental data made with the recently published project [32]. **D** Schematic of stimulation-imaging experiments in acute mouse brainstem slices. NTS nucleus tractus solitarius. **E** Three-dimensional spatiotemporal ΔF/F profiling of an GRAB_NP_ expressing NST neuron to local electrical stimuli. Note one isolated release site indicated by pink arrow in **E**. **F** Pixel-wise maximal ΔF/F plot at the isolated release site indicated by the pink arrow in **E**. Fitting the data points in this plot with a single exponential decay function (pink line) yields an estimated neuropeptide spread length constant of 0.91 µm. Note the collection of exemplary animal experimental data made with the recently published project [32].

Interestingly, the same analysis made with genetically encoded glutamate sensor, iGluSnFR [27], yielded a spread length constant of ~0.60 µm for glutamate (Fig. 2A–C) [32, 80], a slightly smaller value expected for the negatively charged glutamate electrophoretically influenceable by excitatory currents [81]. Moreover, in combination with new GPCR-based genetically encoded neuropeptide sensors, the analysis allowed the first measurements of neuropeptidergic transmission that disclosed a comparable spread length constant of ~0.80 µm (Fig. 2D–F). The identical spatial spread length constants for fast- and slow-acting neurotransmitters across various cells support the notion that synapses optimize their nanoscale pre- and post-synaptic organizational elements (including also the amount of released transmitters, width of synaptic clefts and location of postsynaptic receptors) to maximize efficacy and precision [82–84]. It is attempting to speculate that like the fast-acting neurotransmitters glutamate (e.g., via NMDA receptors) and GABA (e.g., via δ subunit-containing GABA_A_ receptors), neuromodulatory transmitters may employ high affinity receptors and/or cluster releases to achieve certain volume transmission under physiological and pathological conditions. Indeed, accumulating evidence suggests that both fast- and slow-acting neurotransmitters make intercellular communication primarily via highly restricted transmission, with volume transmission serving as a complementary mode under certain conditions [29, 32, 72, 80].

**Fig. 3:**
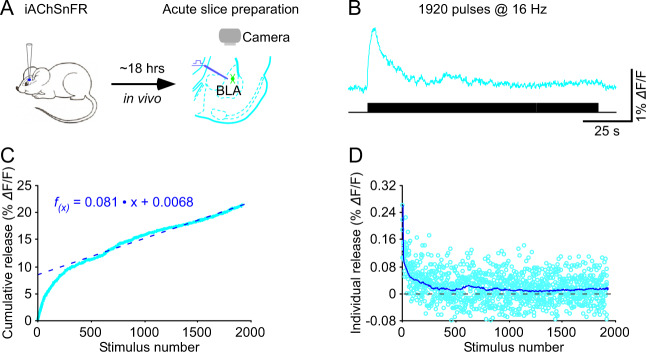
Decoding cholinergic synaptic properties with genetically encoded sensor iAChSnFR. **A** Schematic of stimulation-imaging experiments in ex vivo mouse basolateral amygdala (BLA) preparation. **B** Fluorescence responses of an iAChSnFR expressing amygdalar neuron evoked by a prolonged train of 1920 stimulating pulses at 16 Hz. Plots of cumulative (**C**) and individual (**D**) releases against stimulus numbers enable estimations of the readily releasable pool size, refilling rate, release probability of cholinergic synapses at the amygdalar neuron. Note in **C** a straight cyan line (*f*_(x)_ = 0.081 ● *x* + 0.0068) fitted to the late points of blue cumulative trace with its *y*-axis intercept and slope taken as the pool estimate and vesicle refill rate, respectively, and in **D** cyan trace representing averages of blue individual releases, corresponding to the release probability. Note the collection of exemplary animal experimental data made with the recently published project [32].

A few top-performing genetically encoded neurotransmitter sensors could reliably follow relatively slow neuromodulatory transmission evoked by moderate (actually, more physiological [11]) rates of stimulation over a prolonged period ([32]; our unpublished data) (Fig. 3A, B). Using the previously established vesicle pool models [85–88], one could make reasonably accurate estimations of fundamental neuromodulatory synaptic properties (e.g., release pool size, release probability, and refilling rate). As with the spatial resolution, the temporal resolution of neuromodulatory transmission improves with the increased photon collection. Our preliminary analysis showed that the top-performing genetically encoded neurotransmitter sensors (e.g., iAChSnFR, GRAB_NE1m_, and GRAB_5HT0.5_) emit sufficient amounts of photons in response to endogenous transmitter releases evoked by prolonged trains of stimuli, permitting determination of basic synaptic properties of neuromodulatory transmission at the microscale resolution (Fig. 3C, D). Combining spatial and temporal analysis might even enable decoding of the number of release sites, release probability and quantal size at individual release sites for neuromodulatory transmission ([59]; for an example, see [29, 32]). The pilot analysis suggests that slow neuromodulatory transmission shares some, but not all properties with fast glutamatergic and GABAergic transmission.

### Broad applicability

The new sensor-based superresolution and/or deconvolution microscopic analysis method has broad applicability in various biological and clinical applications. In particular, the method has revealed unanticipated fine control and precision of neuromodulatory transmission [29, 32], which immediately provide explanations to some puzzling clinical observations and suggest new therapeutics for various psychiatric and neurological disorders, including Alzheimer’s disease. For example, the only available therapy for Alzheimer’s disease is based on the finding of diminishing ACh release and deteriorating cholinergic neurons in Alzheimer’s brains—the cholinergic hypothesis [89]. Currently, all FDA-approved Alzheimer’s drugs directly or indirectly inhibit acetylcholinesterase to boost cholinergic signals. These medicines have limited efficacy in cognitive improvement, and upon medication termination, induce irreversible, accelerated deterioration [90, 91]. The fine spatiotemporal control of cholinergic transmission illustrated by genetically encoded sensors sheds light on these two clinical observations [29, 32]. First, acetylcholinesterase inhibitors could reduce the physiological precision of cholinergic transmission (cf. [72, 73]), explaining the limited cognitive improvement. Second, long-term application of acetylcholinesterase inhibitors might homeostatically upregulate acetylcholinesterase levels in Alzheimer’s patients and/or downregulate presynaptic ACh release [91], explaining the accelerated deterioration upon medication termination.

Similarly, dysregulated adrenergic transmission often appears as an early pathological correlate of cognitive decline in Alzheimer’s disease [92, 93]. Genetically encoded sensors revealed a surprising set of adrenergic synaptic properties seemingly designed to run counter to the natural tendency of synapses to achieve fine-tuned linear input-output computation ([32]; our unpublished data). These properties distinguish adrenergic transmission from all other neuronal transmissions, including the fast glutamatergic and GABAergic transmission [12], and other slow neuromodulatory transmission [29, 32]. The findings underscore the unique contribution of adrenergic transmission to fine-tuning of attention [94], optimization of behavior in complex social and physical environments [95], and impairment of complex mental tasks (e.g., reasoning and abstract thinking) in Alzheimer’s patients [96].

Addiction, a leading health problem that results in multiple millions of human disability every year, represents a complex reinforcement behavior manifested by compulsive substance use despite harmful consequence [97]. Addictive disorders involve primary disturbances of the dopaminergic system, although the significance of non-dopaminergic systems, which has been less understood, should not be underestimated [8, 10, 97]. Addictive behavior consists of attention, motivation, and learning processes, which seem to be differentially regulated by distinct local subcellular and rapid subsecond dopaminergic signals [98]. A major weakness of the extant literature is that no study to date has been able to capture the behavior-relevant rapid cell- and subcellular-specific dopaminergic signal dynamics. Genetically encoded DA sensors can (at least in the striatum) qualify dynamics of individual dopaminergic releases with microscopic spatiotemporal resolution [32], and subsequently define synaptic parameters and alternations responsible for specific addictive behavioral events. The insights gained from addictive research should also benefit the understanding of several other related psychiatric and neurological disorders, such as Parkinson’s disease, Huntington’s disease, Tourette’s syndrome, attention-deficit/hyperactivity disorder, and schizophrenia [8].

Stress and adversity responses initiate coordinated neuromodulatory actions at a variety of brain areas to alter attention, anxiety, emotion, pleasure, award, aversion, motor, executive and other behavioral processes, and maladaptive responses may result in melancholic and atypical depressions [99, 100]. Depression affects about one in six individuals in their lifetime and currently, more than 300 million people around the world [99, 101]. The early clinical observation leads to the monoamine hypothesis of depression positing that depressive disorders are due to the decreased monoamines [102]. Today, the monoamine-based antidepressants remain the first line of therapy for depression, yet the treatments have slow onset, low rate of response and low rate of remission (about 30%) as the mechanisms of their action remain elusive [99, 100]. Recent advances in genetic profiling, circuit analysis, and animal model development have unveiled many insights of stress responses and depression, highlighting the heterogeneous subtypes of monoaminergic neurons and circuits involved in various stress and depressive behavioral processes [103, 104]. However, limitations of electrophysiological approach have so far hampered efforts in functionally linking specific transmission changes in specific monoaminergic neurons and circuits with particular stress and depressive behavioral episodes [104]. Genetically encoded neuromodulatory transmitter sensors make it possible to directly delineate specific synaptic alterations of monoaminergic transmission at specific monoaminergic axonal termini of specific subtypes of neurons in specific neuronal circuits [32]. Such analysis should shed new lights on stress responses and depressive disorders.

Sleep is one of the most mysterious yet ubiquitous animal behaviors, and sleep disorders, the most common clinical problems, cause a variety of healthy issues including depression, cognitive decline, immune deficiencies, and obesity [105–107]. ACh and monoamines play complex and central roles in regulation of sleep-wake behaviors. While the early studies reported that neurons in the cholinergic nuclei are essential for initiating and maintaining wakefulness [108, 109], the late results attributed the role in part to glutamatergic neurons in the nuclei. It is still in debate whether cholinergic neurons are necessary for wakefulness [105]. Genetically encoded ACh sensors detected the neuronal activity-evoked large initial and small, sustained ACh releases from cholinergic neurons [29, 32]; these sensors allowed direct assessment of synaptic properties of cholinergic transmission at natural sleep-wake cycles to define cholinergic contributions [105, 106]. Coerulear neurons are not required for wakefulness, but rather crucial for promoting wakefulness under certain conditions [95]. Adrenergic transmission illustrated by genetically encoded NE sensors appeared to operate against the natural tendency of synapses to make a linear input-output computational process ([32]; our unpublished data), ideally for finely tuning wakefulness and attention [94]. This operation, however, would render adrenergic transmission to be vulnerable to system runaway. Adrenergic synapses seem to set a small release pool and a tiny refill rate to ensure neurotransmitter depletion after a certain amount of neuronal activity to create an emergency breakpoint ([32]; our unpublished data), which is presumably responsible for the observed behavioral arrests [94]. Obviously, a comprehensive analysis with genetically encoded NE sensors should demarcate the synaptic mechanisms of adrenergic involvements in sleep-wake cycles and sleep disorders.

Serotonergic and dopaminergic roles in sleep-wake cycles are even less clear. Although some reports suggested that 5HT might initiate and maintain sleep, the others found that serotonergic neurons promoted wake, reflecting presumably the primary and secondary effects of a large variety of serotonergic processes [105, 106]. Defining synaptic alterations of serotonergic transmission at natural sleep-wake cycles with genetically encoded 5HT sensors should provide new insights into serotonergic roles in sleep-wake behaviors [34]. DA is involved in regulation of sleep and wakefulness in a way similar to its other reinforcers (e.g., food, water, and sex) because dopaminergic neuronal activity and extracellular DA levels correlate with circadian oscillations and sleep-orienting behaviors [110, 111]. However, how DA regulates and/or is regulated by the circadian clock and other sleep-wake regulators remain elusive due to the modest DA release changes and varied dopaminergic effects in the midbrain, hypothalamus, and other related brain areas, underlining the importance of decoding synaptic changes of cell type- and projection-specific dopaminergic transmission at the different stages of sleep-wake cycles [105, 106]. Of course, given the modest DA release associated with sleep-wake cycles [106], the next generation of DA sensors with improved fluorescence responses may be required to dissect the synaptic dopaminergic mechanisms underlying sleep-wake behaviors.

**Fig. 4:**
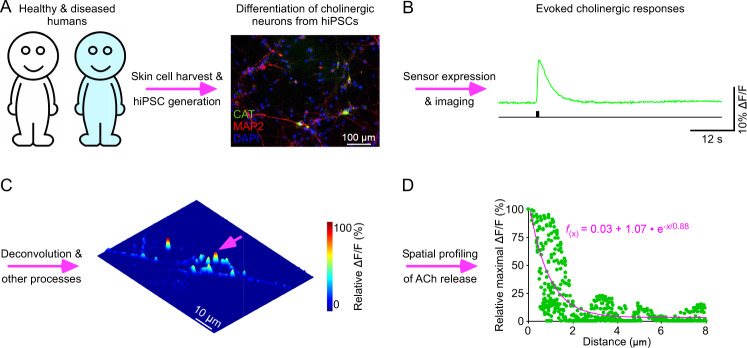
Visualization of human cholinergic transmission with iAChSnFR. **A** Schematic of the differentiation of cholinergic neurons from human-induced pluripotent stem cells (hiPSCs). CAT anti-choline acetyltransferase staining (Abcam, #ab223346), MAP2 anti-microtubule-associated protein 2 staining (Abcam, #ab32454), DAPI 4′,6*-*diamidino*-*2*-*phenylindole nucleic acid staining (Sigma-Aldrich, D9542). Note the authentication of hiPSCs in the previous report [67]. **B** Imaging fluorescence responses of an iAChSnFR expressing human iPSC-derived neuron evoked by a train of 20 stimulating pulses delivered at 32 Hz. **C** Deconvolution microscopic analysis of three-dimensional spatiotemporal ΔF/F profiling of the iAChSnFR expressing human iPSC-derived neuron to local electrical stimuli. Note one isolated release site indicated by pink arrow in **C**. **D** Spatial profiling of the isolated release site indicated by the pink arrow in **C** with a pixel-wise maximal ΔF/F plot. Fitting the data points in this plot with a single exponential decay function (pink line) yields an estimated ACh spread length constant of 0.88 µm.

In summary, genetically encoded sensors have made the experimental verification of the aforementioned hypotheses and possibilities feasible in various disease models. With the human pluripotent stem cell (iPSC) technologies, the diseases models can extend to healthy and diseased human iPSC-derived neuron preparations [112–114]. A pioneering investigation has shown that fast transmission between human iPSC-differentiated neurons behave similarly as that between rodent neurons, with their synaptic properties sharing comparable numerical parameters [115]. This inspired us to express genetically encoded sensors in human cholinergic and monoaminergic neuron culture preparations (Fig. 4A), which are applicable for interrogation of neuromodulatory transmission among human neurons and non-neuronal cells [29, 32, 114]. Micro- and nano-scopic image analysis showed that neuromodulatory transmission in human neuron culture preparations exhibited fine regulation and precision (Fig. 4B–D), reminiscent of that in rodent brain slice and in vivo preparations [29, 32]. These preliminary experiments establish a human-induced neuron system to define synaptic parameters of healthy human neuromodulatory transmission, delineate deficits of diseased human neuromodulatory transmission, screen therapeutic drugs and disease*-*causing genes, and develop potential cell transplantation-based therapies, raising exciting possibilities for regenerative and personalized medicines.
